# Quercetin attenuates AZT-induced neuroinflammation in the CNS

**DOI:** 10.1038/s41598-018-24618-2

**Published:** 2018-04-18

**Authors:** Yi Yang, Xiaokang Liu, Ting Wu, Wenping Zhang, Jianhong Shu, Yulong He, Shao-Jun Tang

**Affiliations:** 1 College of Life Science, Zhejiang Sci-Tech University, Hangzhou, 310018 China; 2 Department of Neuroscience and Cell Biology, University of Texas Medical Branch, Galveston, TX 77555 USA

## Abstract

Highly active anti-retroviral therapy (HAART) is very effective in suppressing HIV-1 replication in patients. However, continuous HAART is required to prevent viral rebound, which may have detrimental effects in various tissues, including persistent neuroinflammation in the central nervous system (CNS). Here, we show that quercetin (3,5,7,3’,4’-pentahydroxy flavones), a natural antioxidant used in Chinese traditional medicines, suppresses the neuroinflammation that is induced by chronic exposure to Zidovudine (azidothymidine, AZT), a nucleoside reverse transcriptase inhibitor (NRTI) that is commonly part of HAART regimens. We found that the up-regulation of pro-inflammatory cytokines and microglial and astrocytic markers induced by AZT (100 mg/kg/day; 8 days) was significantly inhibited by co-administration of quercetin (50 mg/kg/day) in the mouse cortex, hippocampus and spinal cord. We further showed that quercetin attenuated AZT-induced up-regulation of Wnt5a, a key regulator of neuroinflammation. These results suggest that quercetin has an inhibitory effect on AZT-induced neuroinflammation in the CNS, and Wnt5a signaling may play an important role in this process. Our results may further our understanding of the mechanisms of HAART-related neurotoxicity and help in the development of effective adjuvant therapy.

## Introduction

There are over 36.9 million people living with HIV (PLWH) (WHO, 2015). Highly active antiretroviral therapy (HAART) is a standard anti-HIV treatment, which has effectively transformed this previously deadly viral infection into a manageable chronic disease^1^. However, in order to suppress viral replication, PLWH need to stay on HAART continuously.

Neurotoxicity is a critical HAART-related side effect in the nervous system^2–5^. HAART-related neural damage may directly contribute to the disorders that commonly develop in PLWH^6,7^. Hence, effective adjuvant therapy is an urgent need to prevent or reverse HAART-related neurotoxicity. Towards this end, it is important to understand the mechanism by which HAART causes toxicity in the nervous system.

Neuroinflammation, indicated by glial activation and pro-inflammatory cytokine up-regulation, is thought to contribute to the pathogenesis of neurotoxicity via various pathways^2,8–11^. Previous studies showed that compared with naïve PLWH, HAART-treated PLWH had more active microglia in the hippocampus and basal ganglia, indicating a contribution of HAART to neuroinflammation^5^. Consistent with this notion, we recently showed that long-term administration of NRTIs [e.g. zidovudine (AZT), lamivudine (3TC) and stavudine (D4T)], the backbone components of HAART regimens, significantly up-regulated pro-inflammatory cytokines in the mouse CNS^12^.

We are interested in testing the possibility of Chinese traditional medicines as potential HAART adjuvants. Specifically, in this study, we determined the effect of quercetin, a component of Chinese skullcap, on AZT-induced neuroinflammation in the CNS. Quercetin is a natural antioxidant^13^, has anti-neuroinflammatory activity^14–17^, and can inhibit pro-inflammatory cytokine release and microglia activation both *in vivo* and *in vitro*^18–21^. By immunoblotting and immunohistochemistry analyses of pro-inflammatory cytokines (IL-1β and IL-6) and glial markers (GFAP and CD11b) in different CNS regions from mice, we found that quercetin significantly attenuated AZT-induced up-regulation of the pro-inflammatory cytokines and activation of microglia and astrocytes. In addition, we also found that quercetin inhibited AZT-induced up-regulation of Wnt5a, which is an upstream regulator of NRTI-induced neuroinflammation^12^. These results suggest that quercetin is a potential HAART adjuvant that can reverse and attenuate the neurotoxicity caused by AZT and probably other NRTIs.

## Results

### Quercetin inhibits AZT-induced cytokine up-regulation in the CNS

Our previous studies revealed that administration of AZT for 5 days caused the up-regulation of IL-1β, IL-6 and TNF-α in the cortex, the hippocampus and the spinal cord^12^. These cytokines have been implicated in neuroinflammation and inflammation-related diseases^22–31^. Thus, we wanted to test whether quercetin (>95% purity), a natural flavonoid^32–37^ with reported anti-inflammatory activity^38–42^, would inhibit the up-regulation of these cytokines that was induced by a representative NRTI, AZT. C57BL/6 mice (male, 8 weeks) were injected subcutaneously with 100 mg/kg/day AZT for 5 days or 8 days. CNS tissues (cerebral cortices, hippocampi and spinal cords) were collected for Western blotting at the end of the drug treatment periods. The pilot experiments showed that cytokine up-regulation was more obvious at 8 days. Therefore, we treated C57BL/6 mice (male, 8 weeks) with 100 mg/kg/day AZT with or without 50 mg/kg/day quercetin) co-administration for 8 days.

**Figure 1:**
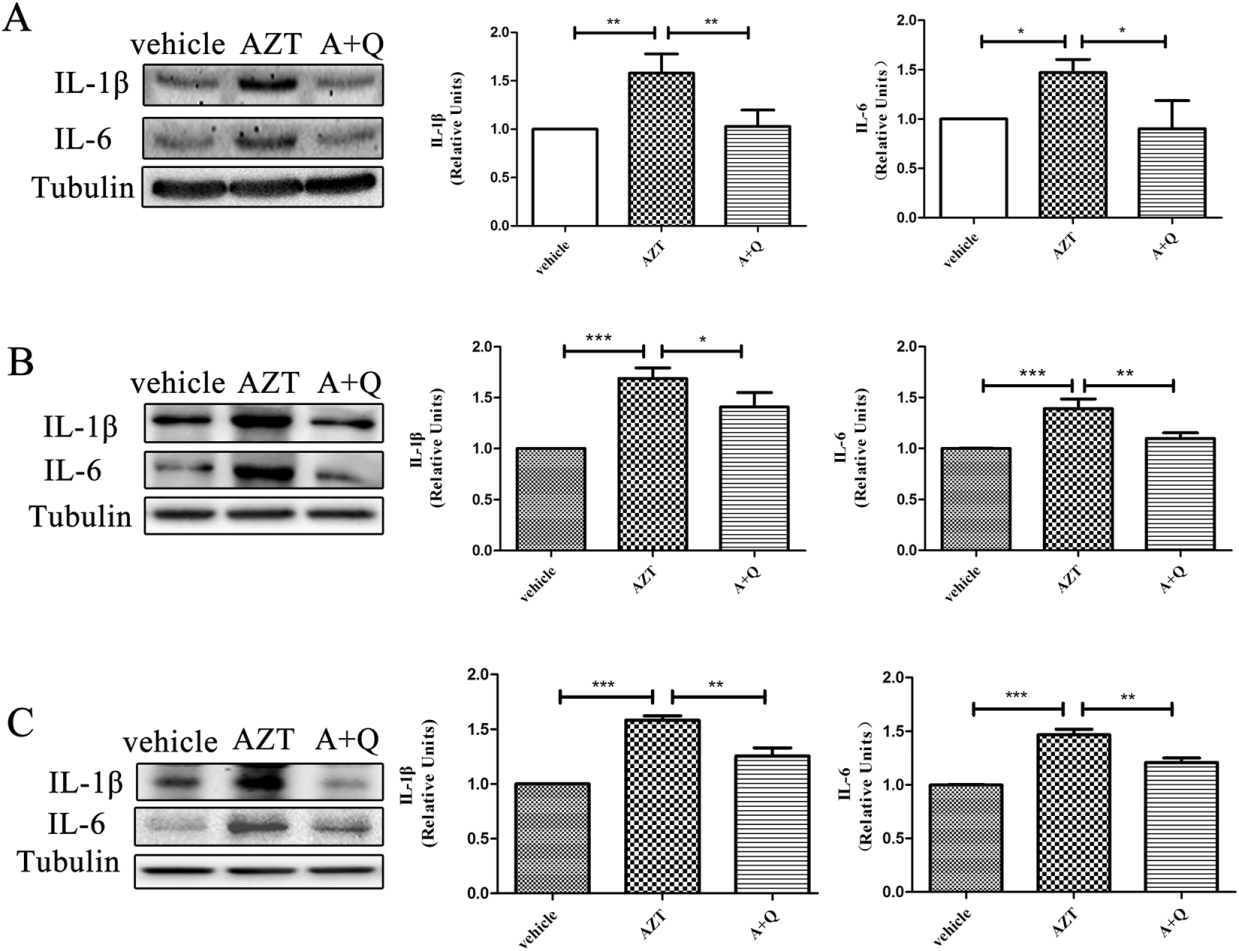
Quercetin inhibited AZT-induced up-regulation of cytokines in the CNS. Expression levels of IL-1β and IL-6 in the cortices (**A**), hippocampi (**B**) and spinal cords (**C**) of mice treated with AZT and quercetin for 8 days. Data are expressed as means ± SEM from at least 8 mice per group (*p < 0.05, **p < 0.01, ***p < 0.001). The cropped blots displayed in the main are presented in full in Supplementary Figure 1.

Western blotting showed that quercetin attenuated AZT-induced IL-1β and IL-6 expression significantly in cerebral cortices, hippocampi and spinal cords (Fig. 1). Most notably among these CNS regions, quercetin completely blocked the cytokine up-regulation in cortices (Fig. 1A). Partial but significant inhibitory effects of quercetin were also observed in hippocampi (Fig. 1B) and spinal cords (Fig. 1C). These data indicate that quercetin can suppress the expression of cytokines induced by AZT in different CNS regions.

### Quercetin attenuates activation of astrocytes induced by AZT in the CNS

**Figure 2:**
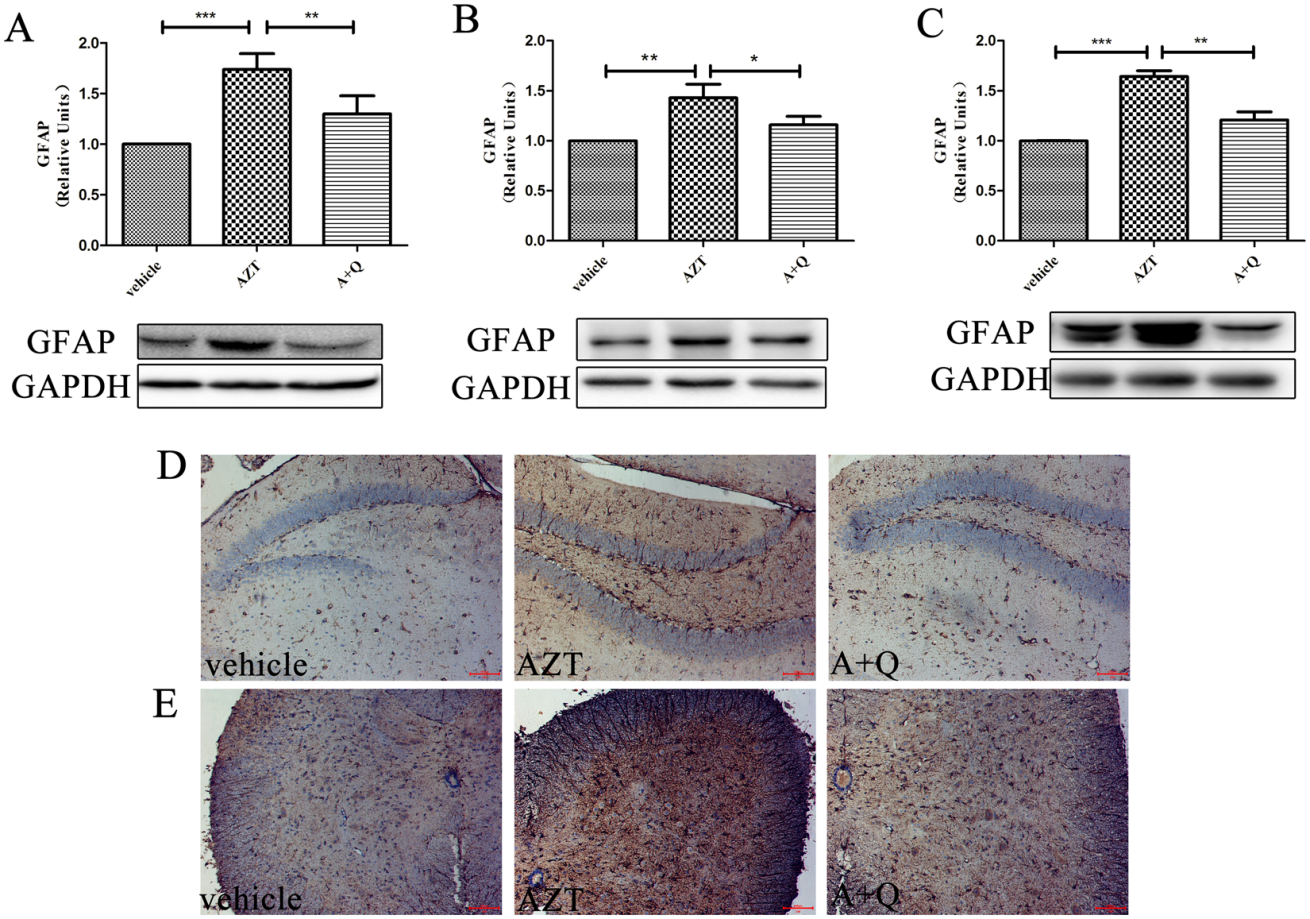
Quercetin attenuates the AZT-induced activation of astrocytes in the CNS. Expression levels of GFAP in the cortices (**A**), hippocampi (**B**) and spinal cords (**C**) of mice treated with AZT and quercetin for 8 days. Data are expressed as means ± SEM from at least 8 mice per group (*p < 0.05, **p < 0.01, ***p < 0.001). Immunocytochemistry staining of GFAP in the hippocampi (**D**) and spinal cords (**E**) of mice treated with AZT and quercetin for 8 days. Scale bars: 100 μm. The cropped blots are presented in full in Supplementary Figure 2.

Activation of astrocytes and microglia is a cellular hallmark of neuroinflammation. AZT has been implicated in astrocyte reaction in PLWH^5^. Reactive astrocytes and microglia are probably the major sources of cytokines in the CNS with neuroinflammation^9,43–46^. We next tested the effect of quercetin on activation of astrocytes and microglia. We observed that the AZT treatment led to significant GFAP up-regulation in the cortex (Fig. 2A), the hippocampus (Fig. 2B) and the spinal cord (Fig. 2C), indicating that AZT induced the astrocyte reaction. Importantly, we also observed that the co-administration of quercetin significantly attenuated the GFAP up-regulation in all of the CNS regions examined (Fig. 2A–C). Immunocytochemistry staining experiments confirmed that AZT treatment led to an increase of GFAP-positive cells in the hippocampus (Fig. 2D) and the spinal cord (Fig. 2E) and that quercetin blocked this increase (Fig. 2D,E). These observations suggest that quercetin inhibits AZT-induced astrocyte activation.

**Figure 3:**
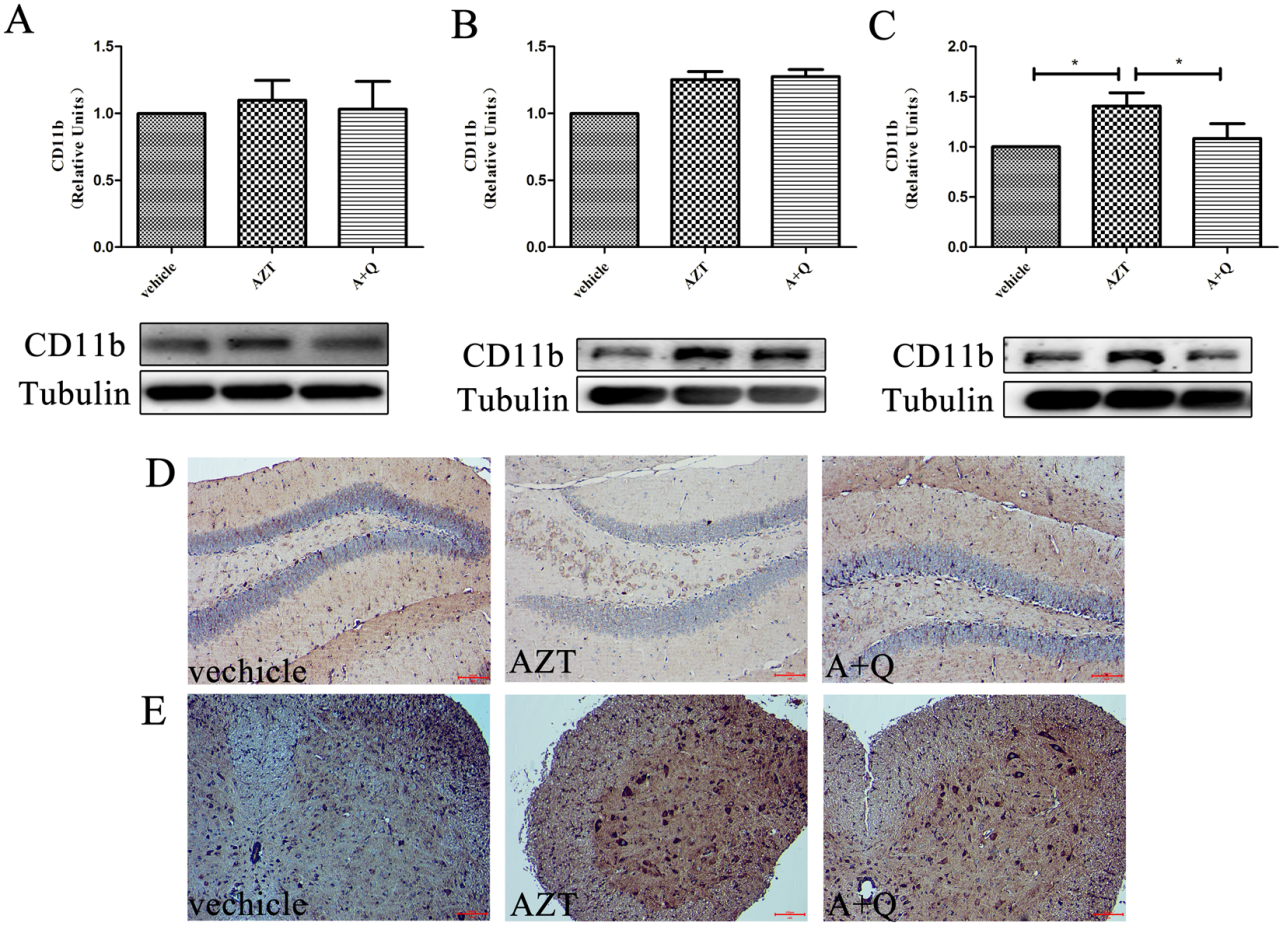
Effects of quercetin on microglia. Expression levels of CD11b in the cortices (**A**), hippocampi (**B**) and spinal cords (**C**) of mice treated with AZT and quercetin for 8 days. Data are expressed as means ± SEM from at least 8 mice per group (*p < 0.05, **p < 0.01, ***p < 0.001). Immunocytochemistry staining of CD11b in the hippocampus (**D**) and the spinal cord (**E**). Scale bars: 100 μm. The cropped blots are presented in full in Supplementary Figure 3.

The analysis of the expression of microglial marker CD11b by immunoblotting (Fig. 3A–C) and immunocytochemistry (Fig. 3D,E) showed that AZT induced significant microglial activation in the spinal cord (Fig. 3C,E) but not in the cortex or the hippocampus (Fig. 3A,B,D). We also observed that quercetin blocked the microglial activation that was induced by AZT in the spinal cord (p < 0.05) (Fig. 3C,E) without affecting the basal level of CD11b expression in the cortex (Fig. 3A) or the hippocampus (Fig. 3B,D).

### Quercetin inhibits Wnt5a up-regulation induced by AZT in the CNS

**Figure 4:**
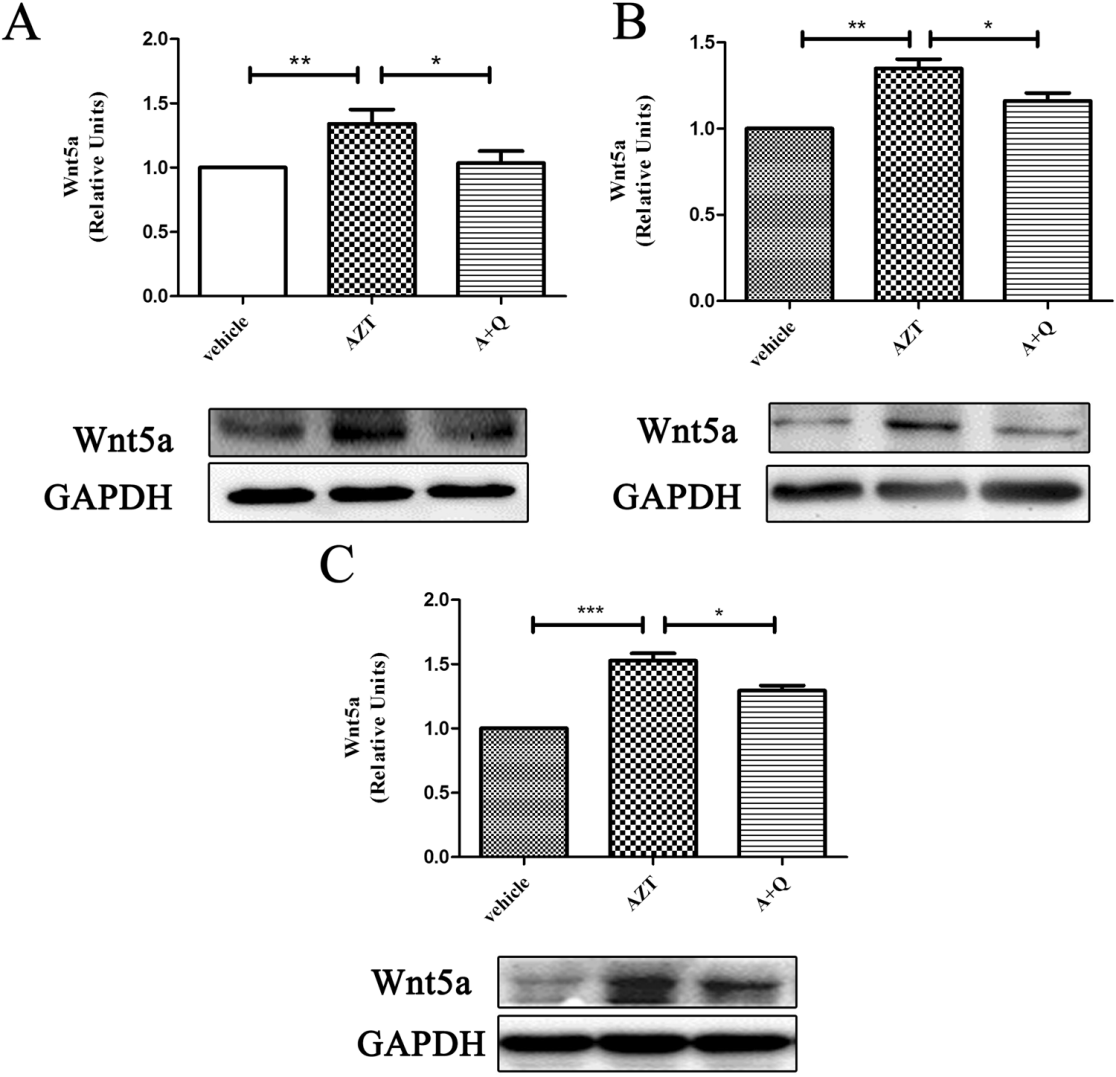
Quercetin inhibits the AZT-induced up-regulation of Wnt5a in the CNS. Expression levels of Wnt5a in the cortices (**A**), the hippocampi (**B**) and the spinal cords (**C**) of mice treated with AZT and quercetin for 8 days. Data are expressed as means ± SEM from at least 8 mice per group (*p < 0.05, **p < 0.01, ***p < 0.001). The cropped blots are presented in full in Supplementary Figure 4.

Previous studies have indicated that Wnt5a is a crucial regulator of neuroinflammation in different experimental systems^47–49^. Because of our recent findings on the NRTI-induced Wnt5a up-regulation in the mouse CNS^12^, we sought to determine the potential effect of quercetin on AZT-induced Wnt5a expression in the CNS. We found that, consistent with the previous report^12^, AZT treatment (8 days) induced Wnt5a up-regulation in cerebral cortices (Fig. 4A), hippocampi (Fig. 4B) and spinal cords (Fig. 4C). In contrast, co-treatment with quercetin significantly attenuated the AZT-induced Wnt5a expression (Fig. 4A–C). These results suggest that quercetin can inhibit the AZT-induced Wnt5a up-regulation in the CNS.

## Discussion

In this study, we tested the effect of quercetin on AZT-induced neuroinflammation in the CNS. Although quercetin was reported to effectively inhibit neuroinflammation in various animal models^16,20,50,51^, our work expanded this inhibitory effect of quercetin to AZT-induced neuroinflammation. Our results show that quercetin inhibits the expression of inflammatory cytokines and astrocyte reaction that are induced by AZT in different CNS regions. Since other NRTIs also cause neuroinflammation^12,52^, it will be interesting to determine the effect of quercetin in other NRTI and non-NRTI HAART components.

It is currently unclear how quercetin inhibits AZT-induced neuroinflammation in the CNS. Quercetin has a broad inhibitory effect on the overexpressed cyclooxygenase-2 and inducible nitric oxide synthase, which are induced by activated astrocytes and microglia, in addition to blocking neuroinflammation^20,53,54^. Furthermore, quercetin inhibits the overexpression of β-catenin and interrupts the canonical Wnt pathway, which has important regulatory roles in neuroinflammation^55–57^. Since we showed previously that NRTIs, including AZT, cause neuroinflammation in the CNS in a Wnt5a signaling-dependent manner^12^, we also looked at Wnt5a expression in this paper. Here, our results indicate that quercetin also attenuates AZT-induced Wnt5a up-regulation (Fig. 4). As the inhibitory effect of quercetin on Wnt5a is similar to that of the Wnt5a antagonist BOX 5^12^, we propose that the mechanisms of quercetin and BOX 5 inhibition on Wnt5a expression are similar. Further experiments will be needed to confirm this. Taken together, we propose that quercetin attenuates AZT-induced neuroinflammation by inhibiting Wnt5a.

Neuroinflammation is implicated in the pathogenesis of various neurological disorders, including neurodegeneration and pathological pain^58–65^. Thus, NRTI-induced neuroinflammation may crucially contribute to the development of neurological deficits in PLWH who are on HAART. Indeed, ddC, an NRTI, is known to cause pain in animal models by a TNF-α-regulated mechanism^66^. Our data indicate that systematic administration of quercetin can effectively suppress AZT-induced neuroinflammation in different CNS regions. This indicates that quercetin can sufficiently cross the blood-brain barrier. Taken together, these findings illustrate the exciting possibility that quercetin may be a potential adjuvant that can be co-administered with HAART to protect the CNS.

## Materials and Methods

### Animals

C57BL/6 mice (male, 8 weeks) were purchased from Shanghai Ling Chang Biological Technology Co., Ltd. Procedures were carried out after the approval from the Zhejiang Sci-Tech University Experimental Animal Ethics Committee, and experiments were in line with relevant standards and regulations to reduce the suffering of the animals.

### AZT and quercetin

AZT (Selleck, S2579) and quercetin (Sigma, Q4951-10G) were dissolved in sterilized PBS to a final concentration of 8 mg/ml and 4 mg/ml, respectively, and stored at −20 °C.

### Subcutaneous (SC) injection

Mice were randomly divided into three groups (n ≥ 8). Group-1 mice were treated with PBS for 8 days (vehicle), group-2 were treated with AZT (100 mg/kg/day) only (AZT), and group-3 were co-treated with quercetin (50 mg/kg/day) and AZT (100 mg/kg/day) (A + Q). The doses of AZT^12^ and quercetin^67–69^ were based on previous studies. SC injection was performed at a volume of 0.25 ml/20 g/day. SC sites were sterilized with 75% alcohol wipes before injection. After injection, gentle pressure was applied to the SC injection site with the alcohol wipe for a moment to prevent the drug from leaking back through the injection site.

### Western blotting and antibodies

The antibodies used are anti-IL-6 (1:2500, Abcam, ab7737); anti-IL-1β (1:5000, R&D, AF-401-NA); anti-Wnt5a (1:2500, Abcam, ab72583); anti-CD11b (1:2500, Abcam, ab133357); anti-GFAP (1:10000, Millipore, MAB360); anti-GAPDH (1:10000, Abcam, ab181602); anti-α-tubulin (1:10000, Proteintech, 66031-1-lg); Goat Anti-Rabbit IgG H&L (HRP) (1:30000, Abcam, ab97051) and Goat Anti-Mouse IgG H&L (HRP) (1:30000, Abcam, ab97023).

### Western blotting and quantification

Mice were anesthetized with inhaled ether and decapitated. Cerebral cortex and hippocampus: Brains were isolated rapidly and then cerebral cortices and hippocampi were dissected carefully. Spinal cord: The whole spines of the mice were exposed, cut near the tail cavity, and pushed out the spinal cord with cold 0.01 M PBS, pH 7.4. Tissues were frozen in liquid nitrogen immediately after collection and stored at −80 °C for western blotting.

Tissues were homogenized in cell lysis buffer (Beyotime, P0013) containing 1 mM PMSF (Sigma, P7626-5G). After centrifugation (12,000 g), the protein concentration in the supernatant was determined with the BCA Protein Assay Kit (BIOMIGA, PW0104) according to manufacturer’s instructions. Boiled total protein (40–60 μg) was loaded on 12% SDS-PA gels. After electrophoresis (120 V for 90–120 min), the protein was blotted onto PVDF membranes (100 V for 90–120 min) at 4 °C. The membrane was blocked with 5% skim milk in TBST (20 mM Tris-HCl, 150 mM NaCl, 0.05% Tween-20) for 2 h at room temperature (RT). Blocked membrane was incubated with primary antibodies in TBST for 2 h at RT. After washing with TBST (3 × 10 min), membranes were incubated with anti-mouse or anti-rabbit secondary antibodies conjugated with HRP in TBST for 1 h at RT and then washed with TBST (3 × 10 min). Protein bands were visualized by Western Bright ECL (Advansta, K-12045-D10) according to the manufacturer’s instructions. GAPDH or tubulin was blotted as a loading control.

### Immunohistochemistry

Mice were anesthetized with inhaled ether and perfused transcardially with PBS. Brains were isolated rapidly, and spinal cords were dissected as above. Tissues were fixed in paraformaldehyde (PFA; 4% in 0.1 M PBS) for 40 min, and paraffin sections were prepared as usual.

Paraffin sections were deparaffinized in xylene and rehydrated in ddH_2_O with different concentrations of ethanol (100%, 95%, 80%, and 70%). Deparaffinized sections were treated with 0.3% H_2_O_2_ to block endogenous peroxidase. After incubation with EDTA buffer (pH 8.0) to repair antigen (95–100 °C for 20 min), the sections were incubated with anti-GFAP (1:800) or anti-CD11b (1:200) in 1% BSA for 1 h at RT. Then, the sections were incubated with secondary antibody for 30 min and stained with DAB (Gene Technology). Finally, the sections were rinsed first with hematoxylin and then with ddH_2_O, dehydrated and mounted for imaging.

## Electronic supplementary material

Supplementary Information
