# Supplementary material for: Quercetin attenuates AZT-induced neuroinflammation in the CNS
Source: Sci Rep. 2018 Apr 18;8:6194. doi: 10.1038/s41598-018-24618-2 (PMC5906611; doi:10.1038/s41598-018-24618-2)

Supplementary Information

Quercetin attenuates AZT-induced neuroinflammation in the CNS

Yi Yang[1+], Xiaokang Liu[1+], Ting Wu[1], Wenping Zhang[1], Jianhong Shu[1,*], Yulong He[1,*], Shao-Jun Tang[1,2]

1.College of Life Science, Zhejiang Sci-Tech University, Hangzhou 310018, China

2.Department of Neuroscience and Cell Biology, University of Texas Medical Branch, Galveston, TX 77555, USA

*Corresponding author: Yulong He, Tel./Fax.: +86-0571-8684-3193, E-mail: heyulong2003@163.com

*Corresponding author: Jianhong Shu, Tel./Fax.: +86-0571-8684-3199, E-mail: shujianhong@zstu.edu.cn

+These authors contributed equally to this work

Supplementary Figure 1. Full blots of Fig. 1A, 1B, and 1C

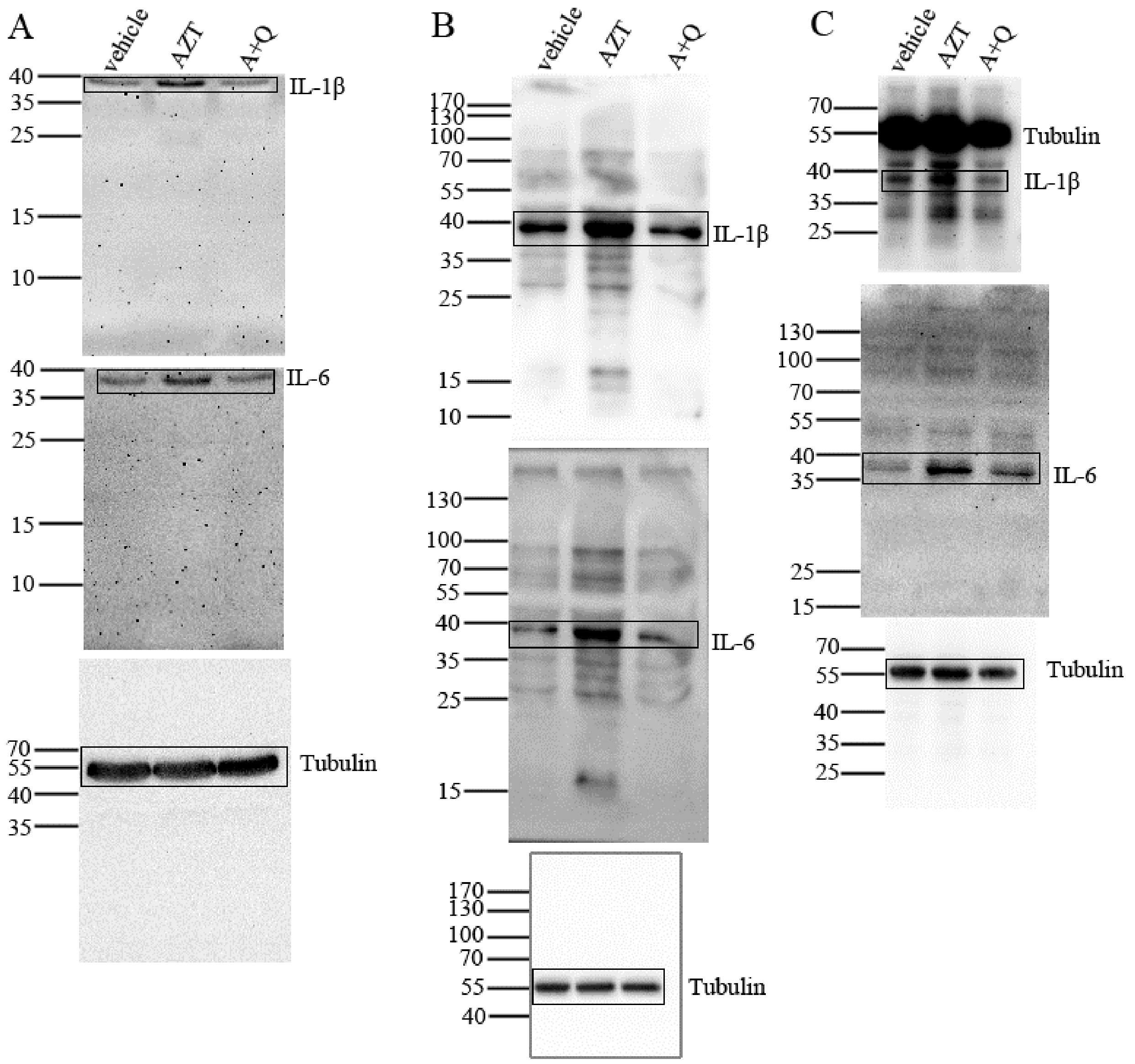

Supplementary Figure 2. Full blots of Fig. 2A, 2B, and 2C

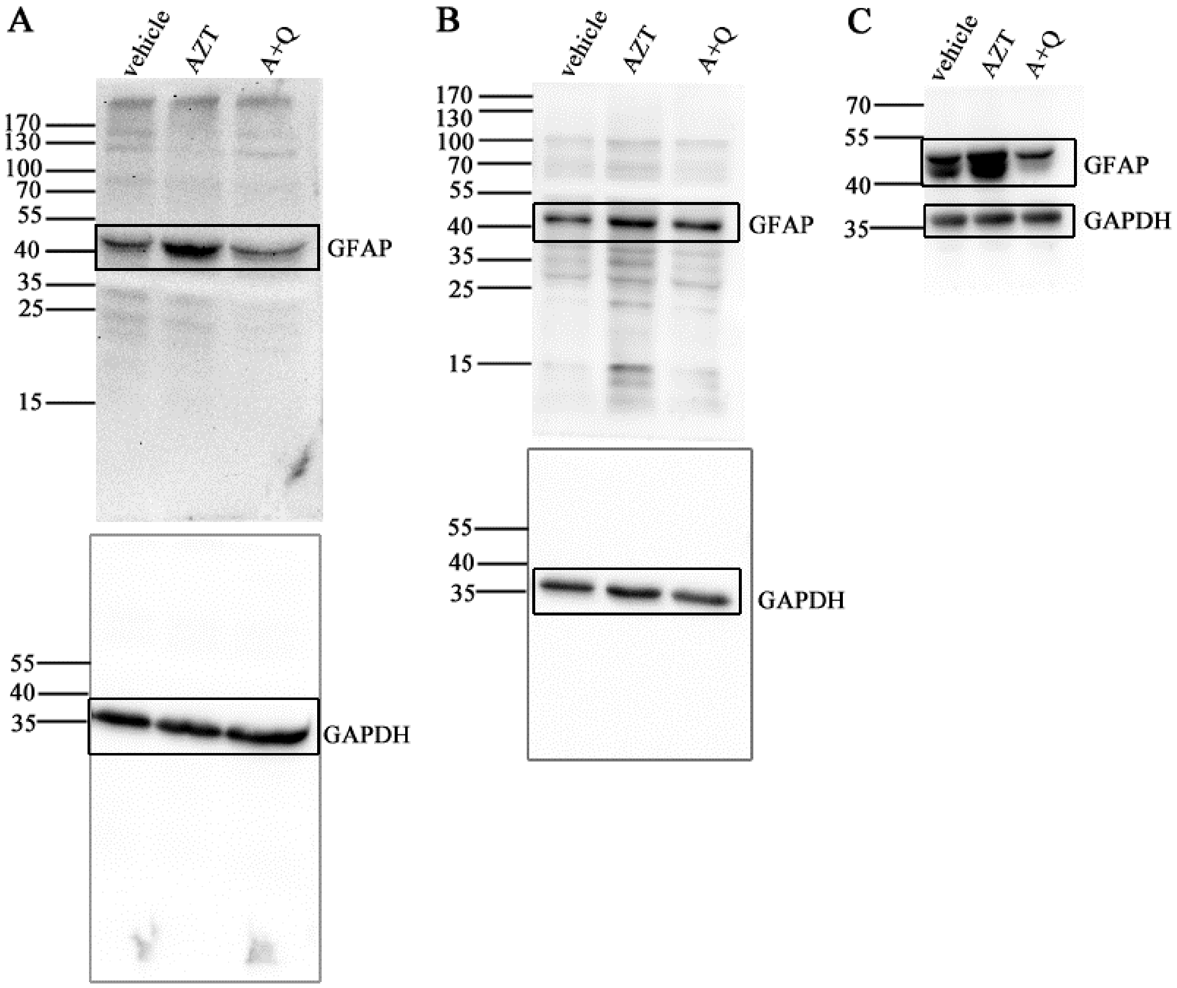

Supplementary Figure 3. Full blots of Fig. 3A, 3B, and 3C

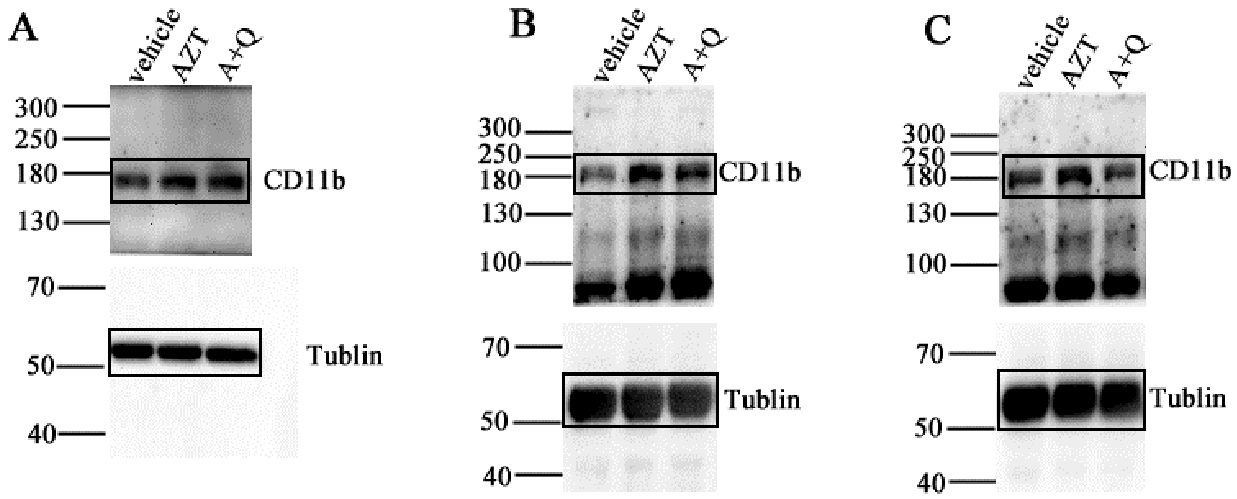

Supplementary Figure 4. Full blots of Fig. 4A, 4B, and 4C

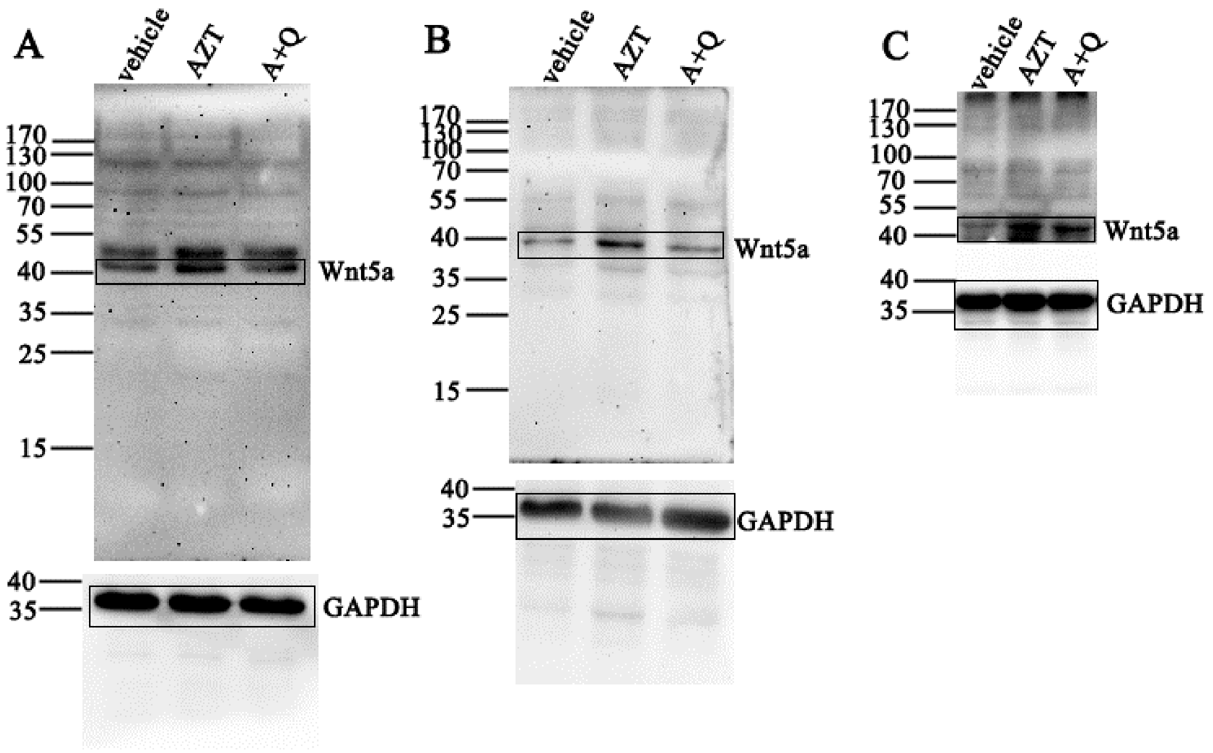

Supplement: Supplementary file 1 — Supplementary Information [file 41598_2018_24618_MOESM1_ESM.pdf]
